# 3-way Networks: Application of Hypergraphs for Modelling Increased Complexity in Comparative Genomics

**DOI:** 10.1371/journal.pcbi.1004079

**Published:** 2015-03-27

**Authors:** Deborah A Weighill, Daniel A Jacobson

**Affiliations:** 1 Institute for Wine Biotechnology, Stellenbosch University, Stellenbosch, South Africa; 2 Comparative Genomics Group, Biosciences Division, Oak Ridge National Laboratory, Oak Ridge, Tennessee, United States of America; 3 Bredesen Center for Interdisciplinary Research and Graduate Education, University of Tennessee, Knoxville, Tennessee, United States of America; Hellas, Greece

## Abstract

We present and develop the theory of 3-way networks, a type of hypergraph in which each edge models relationships between triplets of objects as opposed to pairs of objects as done by standard network models. We explore approaches of how to prune these 3-way networks, illustrate their utility in comparative genomics and demonstrate how they find relationships which would be missed by standard 2-way network models using a phylogenomic dataset of 211 bacterial genomes.

## Author Summary

Genomes contain the information underlying the molecular functions of an organism. One way to compare the entire genomes of different organisms is to compare their gene-family content profiles which is effectively a comparison of their functional potential. Standard networks, when used to model phylogenomic similarities, are not capable of capturing some of the underlying complexity of the relationships between genomes. In order to address this, we have developed a new three-way similarity metric and constructed three-way networks modelling the relationships between 211 bacterial genomes. We find that such three-way networks find cross-species genomic similarities that would have been otherwise missed by simpler models such as standard networks.

## Introduction

Network models are a useful reductionist approach for modelling complex systems. Networks involve representing a collection of objects as nodes, and representing relationships between those objects as edges. Thus networks model a system in a pairwise manner, breaking a system down into individual parts (nodes), modelling relationships between pairs of these individual parts (edges) and then reconstructing the system as a network [1]. However, modelling a system based on only pairwise relationships biases the model against more complex relationships that may exist in the system. To this end, we introduce a new ternary network definition, namely 3-way networks based on the concept of hypergraphs. A Hypergraph is a generalized network, in which an edge can model the relationship between an arbitrary number of objects [2, 3]. Clustering algorithms for hypergraphs, also known as Hypergraph Partitioning algorithms, have been developed in which nodes of a graph are assigned to *k* partitions. This can be performed by minimising the net cut, which is defined as the number of hyperedges which connect nodes in different partitions [3]. Software packages such as hMetis [4] are available to perform this *k*-way clustering.

In this work, we use 3-way networks to model the relationships between triplets of objects instead of pairs of objects. The concept of calculating the similarity between objects three at a time is not a novel concept [5–7] and general hypergraphs [2] have previously been used in certain areas of biology, including metabolic modelling, gene expression and RNA interaction studies [8–12]. However, to our knowledge, this is the first time that the concept of 3-way networks has been applied in the field of comparative genomics.

In this study, we develop the theory around 3-way networks in terms of abstract definition, weighting 3-way networks and pruning 3-way networks. We develop a new 3-way metric for the weighting of 3-way edges. We then apply a 3-way network model to a set of 211 bacterial genomes, modelling the similarities between the bacteria on a whole genome scale, (based on gene family content), and compare the resulting 3-way networks to those obtained using standard 2-way network models.

## Results/Discussion

### Definition of 3-way Networks

A network, or graph, *G* is an ordered pair, defined as

$$\begin{array}{c} G=(V,E) \end{array}$$ (1)

where *V* = {*v*_1_, *v*_2_, …, *v*_*n*_} is a set of *n* nodes and *E* = {*e*_1_, *e*_2_, …, *e*_*m*_} is a set of *m* edges [13]. In this case, nodes represent a certain set of objects of interest and edges can be interpreted as relationships between these objects. In particular, edges represent pairwise relationships and thus are defined (for an undirected network) as pairs of nodes. For clarity, we refer to these networks as 2-way networks because of the pairwise nature of the edges. With the aim of modelling higher order relationships than simply pairwise relationships, we define 3-way networks as network models of ternary relationships, i.e. relationships between triplets of objects. 3-way networks are defined by replacing the previous definition of an edge as a set of 2 nodes by a set of 3 nodes. Thus a 3-way network is a type of hypergraph [2]. This can be formalized with the following definition:

**Definition 1.** A 3-way network is a graph *G* = (*V*, *E*) where *V* = {*v*_1_, *v*_2_, …, *v*_*n*_} is the set of nodes and *E* = {*e*_1_, *e*_2_, …, *e*_*m*_} is the set of edges. Each edge *e*_*i*_ is defined as a set of 3 nodes, *e*_*i*_ = {*v*_*x*_, *v*_*y*_, *v*_*z*_} where *x*, *y*, *z* ∈ {1, 2, 3, …, *m*}.

**Fig 1:**
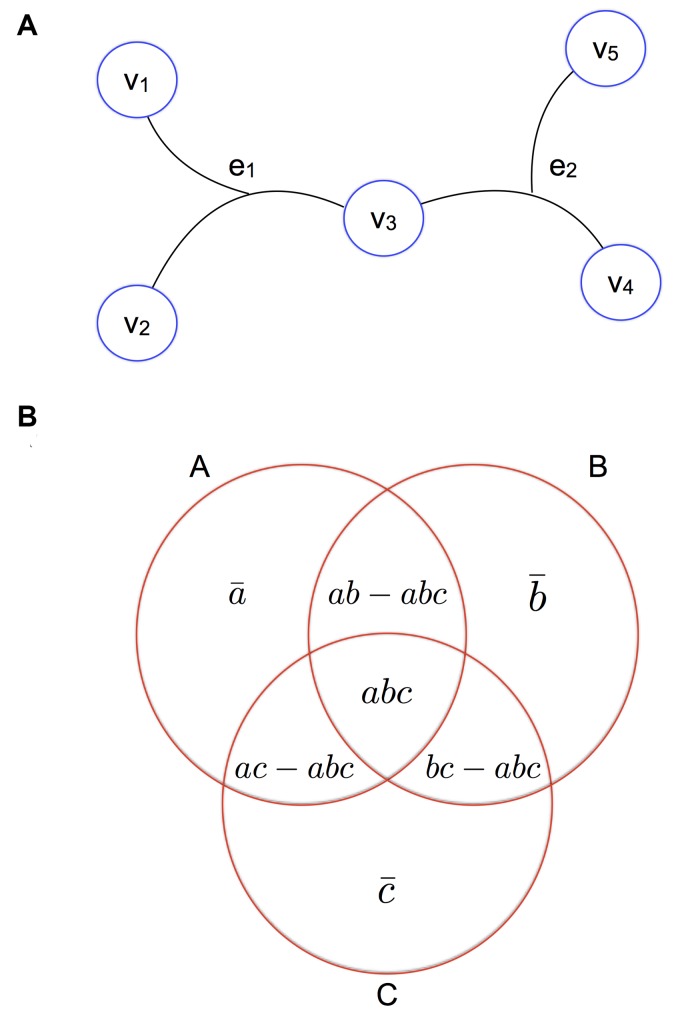
3-way edges and intersections. (a) A small, 3-way network consisting of 5 nodes *v*_1_, *v*_2_, *v*_3_, *v*_4_ and *v*_5_ and two 3-way edges *e*_1_ and *e*_2_. Edge *e*_1_ connects nodes *v*_3_, *v*_4_ and *v*_5_ and edge *e*_2_ connects nodes *v*_1_, *v*_2_ and *v*_3_. (b) Venn diagram for a 3-way intersection of species. *a* is the number of families present in species *A*, *b* is the number of families present in species *B*, *c* is the number of families present in species *C*, *ab* is the number of families present in species *A* and species *B*, *ac* is the number of families present in species *A* and species *C*, *bc* is the number of families present in species *B* and species *C*, *abc* is the number of families present in species *A*, *B* and *C*, $\bar{a}$ is the number of families present only in species *A*, $\bar{b}$ is the number of families present only in species *B* and $\bar{c}$ is the number of families present only in species *C*.

Graphically, each 3-way edge is a line connecting 3 nodes, which can be interpreted as a relationship between 3 objects. An example of a 3-way network with 5 nodes, *V* = {*v*_1_, *v*_2_, *v*_3_, *v*_4_, *v*_5_} and 2 edges, *E* = {*e*_1_, *e*_2_} = {{*v*_1_, *v*_2_, *v*_3_}, {*v*_3_, *v*_4_, *v*_5_}} is shown in Fig. 1a.

### Weighted 3-way Networks

#### 3-way Sørensen Index

In a 2-way network, each edge can be assigned a weight indicating the strength of the relationship between the two nodes the edge is connecting. This concept can easily be extended to a 3-way network, in which an edge weight will indicate the strength of the relationship between the 3 nodes the edge is connecting. For a 3-way network, this requires a similarity metric which quantifies the similarity between 3 objects at a time. Assuming that each object is represented by a vector, a similarity metric which quantifies the similarity between 3 vectors is needed. The Sørensen Index [14] is a similarity metric which quantifies the overlap between the features of pairs of objects. Let *A* and *B* be two objects and let each object be viewed as a set of features. The Sørensen Index *S*_2_(*A*, *B*) is defined as:

$$\begin{array}{c} S_{2}\left(A,B\right)=\frac{2ab}{a+b} \end{array}$$ (2)

where *a* is the number of features of object *A*, *b* is the number of features of object *B* and *ab* is interpreted as the number of features shared by object *A* and object *B* [15]. If objects are represented by vectors, the Sørensen Index between two vectors *X* and *Y* can be expressed as:

$$\begin{array}{c} S_{2}\left(X,Y\right)=\frac{2\sum_{i}\min\left({X_{B}}_{i},{Y_{B}}_{i}\right)}{\sum_{i}\left({X_{B}}_{i}+{Y_{B}}_{i}\right)} \end{array}$$ (3)

where *X*_*B*_ and *Y*_*B*_ are binary vectors defined as:

$$\begin{array}{c} {X_{B}}_{i}=\left\{\begin{array}{cc} 1 & \text{if}\;X_{i}\geq 1\\ 0 & \text{if}\;X_{i}=0 \end{array}\right. \end{array}$$ (4)

$$\begin{array}{c} {Y_{B}}_{i}=\left\{\begin{array}{cc} 1 & \text{if}\;Y_{i}\geq 1\\ 0 & \text{if}\;Y_{i}=0 \end{array}\right. \end{array}$$ (5)

An extension of the Sørensen Index exists for calculating the similarity between triplets of objects. This metric was originally developed for quantifying the similarity between the species content of different biological samples [5]. Generally, for each triplet of objects *A*, *B*, and *C*, each represented by a vector, the three-way Sørensen index can be defined as:

$$\begin{array}{c} S_{3}\left(ABC\right)=\frac{3}{2}\left(\frac{ab+ac+bc-abc}{a+b+c}\right) \end{array}$$ (6)

where *a* is the number of features present in object *A*, *b* is the number of features present in object *B*, *c* is the number of features present in object *C*, *ab* is the number of features present in object *A* and object *B*, *ac* is the number of features present in object *A* and object *C*, *bc* is the number of features present in object *B* and object *C* and *abc* is the number of features present in object *A*, *B* and *C* [5]. These variables can be visualized on a venn diagram (Fig. 1b).

The 3-way Sørensen Index can also be expressed in vector format as follows:

$$\begin{array}{c} S_{3}\left(X,Y,Z\right)=\frac{\frac{3}{2}\sum_{i}\left(\min\left({X_{B}}_{i},{Y_{B}}_{i}\right)+\min\left({X_{B}}_{i},{Z_{B}}_{i}\right)+\min\left({Y_{B}}_{i},{Z_{B}}_{i}\right)-\min\left({X_{B}}_{i},{Y_{B}}_{i},{Z_{B}}_{i}\right)\right)}{\sum_{i}\left({X_{B}}_{i}+{Y_{B}}_{i}+{Z_{B}}_{i}\right)} \end{array}$$ (7)

#### 3-way Czekanowski Index

A quantitative version of the Sørensen Index is called the Czekanowski Index [16]. For two vectors *X* and *Y*, the Czekanowski Index is defined as:

$$\begin{array}{c} C_{2}\left(X,Y\right)=\frac{2\sum_{i}\min\left(X_{i},Y_{i}\right)}{\sum_{i}\left(X_{i}+Y_{i}\right)} \end{array}$$ (8)

Notice that the equation is the same as that of the Sørensen Index in vector format, except that the original vectors are used and not binary vectors. The Czekanowski Index thus considers the size of the overlaps between features of an object and not simply the presence or absence of features. Using the same structure as the 3-way Sørensen Index, we extended the Czekanowski Index to a 3-way form. For 3 vectors *X*, *Y* and *Z*, we have defined the 3-way Czekanowski Index between the three vectors as:

$$\begin{array}{c} C_{3}\left(X,Y,Z\right)=\frac{\frac{3}{2}\sum_{i}\left(\min\left(X_{i},Y_{i}\right)+\min\left(X_{i},Z_{i}\right)+\min\left(Y_{i},Z_{i}\right)-\min\left(X_{i},Y_{i},Z_{i}\right)\right)}{\sum_{i}\left(X_{i}+Y_{i}+Z_{i}\right)} \end{array}$$ (9)

### Pruning 3-way Networks

Many approaches used to prune edges from a network such as Maximum Spanning Tree (MST) algorithms and clustering algorithms are designed for 2-way networks and are not directly applicable to 3-way networks. However, certain approaches are easily transferable to 3-way networks, namely thresholding and best-edge selection.

#### Thresholding

Thresholding can easily be transferred from a 2-way network to a 3-way network. Thresholding is one of the simplest ways to prune any network. A threshold is set and edges with a weight below the chosen threshold are removed. In order to determine a justifiable threshold for a 3-way Sørensen network we have developed the following theorem:

**Theorem 1.** If the intersection of three objects *abc* is zero (i.e. there is no feature present in all three objects), then $S_{3}(ABC)\leq \frac{3}{4}$.

*Proof.* If *abc* = 0, then

$$\begin{array}{ccc} S_{3}\left(ABC\right) & = & \frac{3}{2}.\frac{ab+ac+bc-abc}{a+b+c}\\ & = & \frac{3}{2}.\frac{ab+ac+bc-abc}{2\left(ab+ac+bc\right)+\bar{a}+\bar{b}+\bar{c}}\\ & = & \frac{3}{2}.\frac{ab+ac+bc}{2\left(ab+ac+bc\right)+\bar{a}+\bar{b}+\bar{c}}. \end{array}$$

where $\bar{a}$, $\bar{b}$, and $\bar{c}$ are defined in Fig. 1b. There are two cases to consider.

Case 1: If $\bar{a}$, $\bar{b}$ and $\bar{c}$ are all equal to 0, then

$$\begin{array}{ccc} S_{3}\left(ABC\right) & = & \frac{3}{2}.\frac{ab+ac+bc}{2\left(ab+ac+bc\right)+\bar{a}+\bar{b}+\bar{c}}\\ & = & \frac{3}{2}.\frac{ab+ac+bc}{2(ab+ac+bc)}\\ & = & \frac{3}{2}.\frac{1}{2}\\ & = & \frac{3}{4}. \end{array}$$

Thus if *abc* = 0 and $\bar{a}$, $\bar{b}$ and $\bar{c}$ are all equal to 0 then $S_{ABC}=\frac{3}{4}$.

Case 2: If $\bar{a}$, $\bar{b}$ and $\bar{c}$ are all greater than zero 0 (they cannot be less than zero, since there cannot be a negative number of features associated with an object), then

$$\begin{array}{ccc} 2\left(ab+ac+bc\right)+\bar{a}+\bar{b}+\bar{c} & > & 2(ab+ac+bc)\\ \text{Therefore,}\;S_{3}\left(ABC\right) & = & \frac{3}{2}.\frac{ab+ac+bc}{2\left(ab+ac+bc\right)+\bar{a}+\bar{b}+\bar{c}}\\ & < & \frac{3}{2}.\frac{ab+ac+bc}{2(ab+ac+bc)}\\ & = & \frac{3}{4} \end{array}$$

Thus if *abc* = 0 and $\bar{a}$, $\bar{b}$ and $\bar{c}$ are all greater than zero 0, $S_{3}(ABC)<\frac{3}{4}$. Combining these two cases, we can conclude that if *abc* = 0, $S_{3}(ABC)\leq \frac{3}{4}$. This implies that, for a given 3 species, there are no gene families present in only one of the 3 species, then the 3-way Sørensen Index between the 3 species will be greater than 0.75.

A similar thresholding strategy can be adopted for the 3-way Czekanowski Index. We need the following:

**Lemma 1.** Given integers *a*, *b* and *c*, the following relation holds:

$$\begin{array}{c} \min(a,b)+\min(a,c)-\min(a,b,c)\leq a \end{array}$$ (10)

We now prove a theorem similar to Theorem 1, but relating to the 3-way Czekanowski Index.

**Theorem 2.** Given 3 species *X*, *Y*, and *Z*, if there is no gene family present in all 3 species, then $C_{3}(XYZ)\leq \frac{3}{4}$.

*Proof.* If there is no gene family present in all 3 species *X*, *Y* and *Z*, then ∑_*i*_min(*X*_*i*_, *Y*_*i*_.*Z*_*i*_) = 0. Therefore,

$$\begin{array}{ccc} C_{3}\left(X,Y,Z\right) & = & \frac{\frac{3}{2}\sum_{i}\left(\min\left(X_{i},Y_{i}\right)+\min\left(X_{i},Z_{i}\right)+\min\left(Y_{i},Z_{i}\right)-\min\left(X_{i},Y_{i},Z_{i}\right)\right)}{\sum_{i}\left(X_{i}+Y_{i}+Z_{i}\right)}\\ & = & \frac{\frac{3}{2}\sum_{i}\left(\min\left(X_{i},Y_{i}\right)+\min\left(X_{i},Z_{i}\right)+\min\left(Y_{i},Z_{i}\right)\right)-\sum_{i}\left(\min(X_{i},Y_{i},Z_{i})\right)}{\sum_{i}\left(X_{i}+Y_{i}+Z_{i}\right)}\\ & = & \frac{\frac{3}{2}\sum_{i}\left(\min\left(X_{i},Y_{i}\right)+\min\left(X_{i},Z_{i}\right)+\min\left(Y_{i},Z_{i}\right)\right)}{\sum_{i}\left(X_{i}+Y_{i}+Z_{i}\right)} \end{array}$$

Using Lemma 1, this can be expanded as:

$$\begin{array}{ccc} C_{3}\left(X,Y,Z\right) & = & \frac{\frac{3}{2}\sum_{i}\left(\min\left(X_{i},Y_{i}\right)+\min\left(X_{i},Z_{i}\right)+\min\left(Y_{i},Z_{i}\right)\right)}{\sum_{i}\left(X_{i}+Y_{i}+Z_{i}\right)}\\ & \leq & \frac{\frac{3}{2}\sum_{i}\left(\min\left(X_{i},Y_{i}\right)+\min\left(X_{i},Z_{i}\right)+\min\left(Y_{i},Z_{i}\right)\right)}{2\sum_{i}\left(\min\left(X_{i},Y_{i}\right)+\min\left(X_{i},Z_{i}\right)+\min\left(Y_{i},Z_{i}\right)\right)}\\ & = & \frac{3}{4} \end{array}$$

Thus if ∑_*i*_min(*X*_*i*_, *Y*_*i*_.*Z*_*i*_) = 0, then $C_{3}(X,Y,Z)\leq \frac{3}{4}$.

Thus the minimum justifiable threshold for 3-way Sørensen and 3-way Czekanowski networks is 0.75.

#### Best edges

Another simple way to prune a network is to select for each node, the best *x* edges connected to that node, i.e. select the *x* edges with the highest weight for each node. This is easily done by taking a list of all edges connected to a given node, ranking them by weight from highest to lowest, and then selecting the top *x* edges. This approach does not depend on the definition of the edge. It is directly transferable from the concept of a 2-way network to the concept of a 3-way network.

### Phylogenomic Networks of Bacterial Genomes

Gene families were calculated across a dataset consisting of 211 bacterial genomes using TribeMCL [17] and gene family content profiles constructed for each bacterial species. Various phylogenomic 2-way similarity, 3-way similarity and gene family enrichment networks were then constructed in order to investigate the relationships between the bacterial species based on gene family content and to compare the effect of 3-way networks as opposed to 2-way networks. These networks are described below. In each network, nodes represent bacterial species and edges represent similarities between species based on 2-way or 3-way similarity between their gene family content profiles, or represent connections between species based on shared gene family enrichment.

#### 3-way and 2-way Sørenesen networks

**Fig 2:**
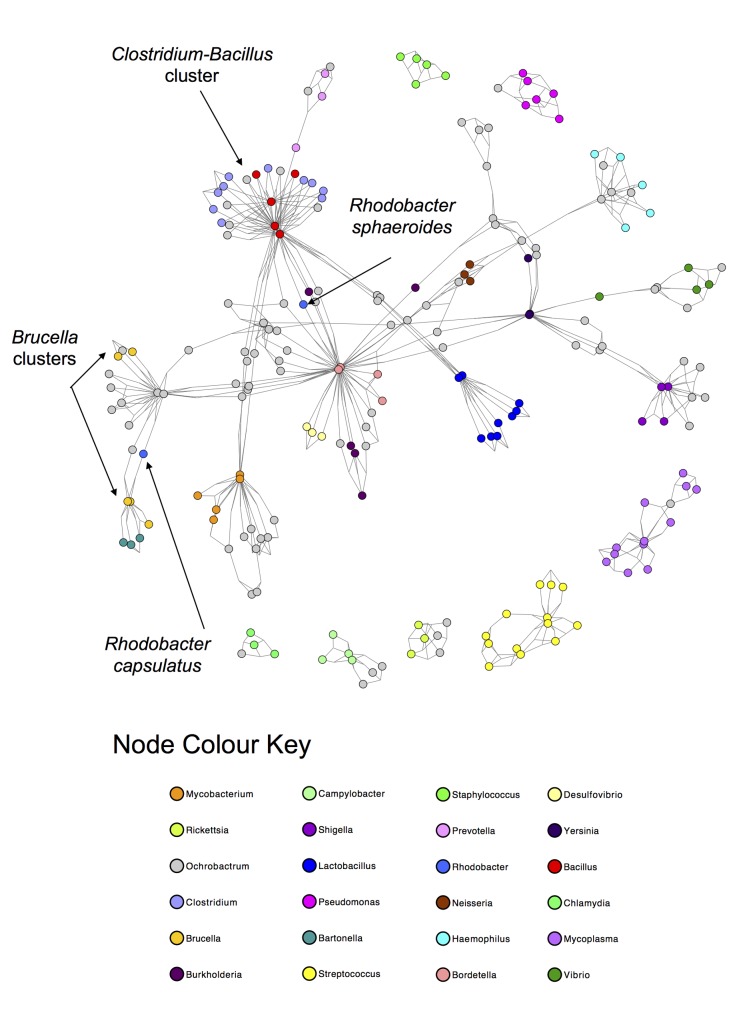
Best-Edges 3-way Sørensen Network. 3-way Sørensen network pruned by selecting the best and second best edge for each node. Nodes represent bacterial species and edges represent similarity between triplets of bacterial species based on gene family content, quantified using the 3-way Sørensen Index. Nodes are coloured according to genus. Default colour is grey.

The concept of 3-way networks was developed in order to attempt to model more complex relationships that would otherwise be missed by pairwise relationships. To this end, the definition of an edge was extended to represent a ternary relationship, i.e. a relationship between 3 nodes. In order to quantify these ternary relationships, a 3-way similarity metric was chosen, namely the Sørensen Index. This allowed “high order similarities” or similarities between more than two species to contribute to our interpretation. The 3-way Sørensen Index was used to quantify the similarity between all triplets of bacterial species, based on their gene family content. Applying a threshold of 0.76 allowed us to select for edges which we were sure had a contributing factor of the 3-way intersection and not simply a high intersection between pairs of species (See Theorem 1). This thresholded network can be seen in S1 Fig. Large coloured nodes represent bacterial species and the combination of the small white nodes and the grey 2-way edges represent 3-way edges. Certain genera were selected and those bacterial species nodes coloured according to genus. (The default node colour was grey, thus grey nodes are not all in the same genus). The 3-way network was also pruned by selecting only the best and second best edge for each node. This best-edge 3-way Sørensen network can be seen in Fig. 2.

**Fig 3:**
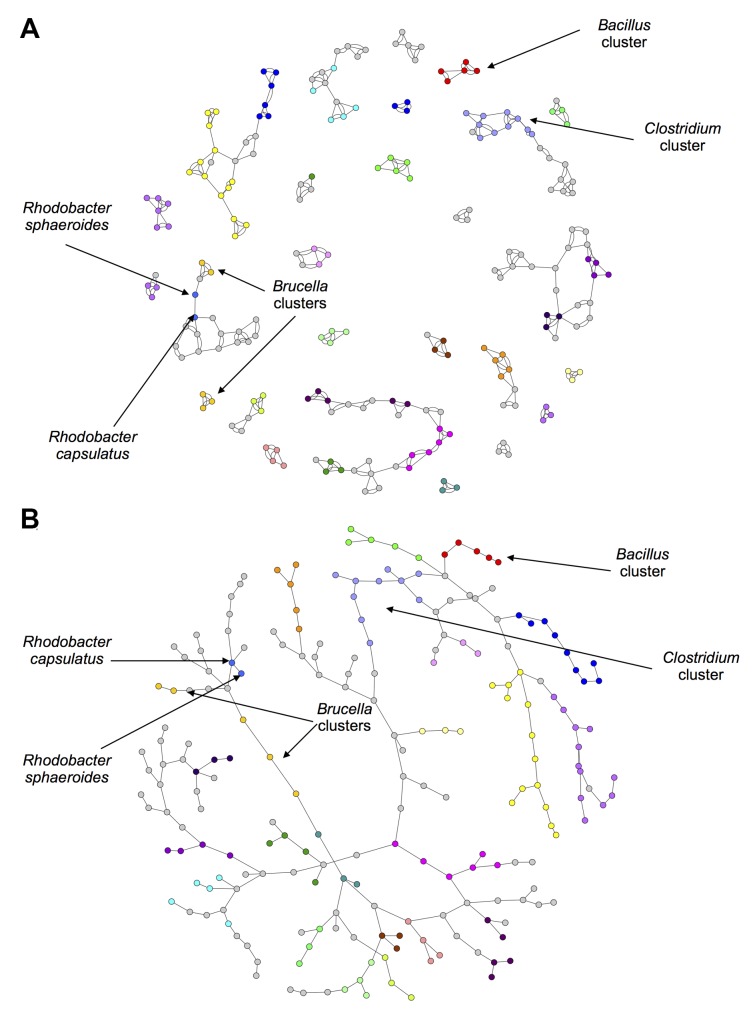
2-way Sørensen Networks. (a) 2-way Sørensen Best Edges Network (b) Maximum Spanning Tree (MST) of the all-vs-all Sørensen network. Nodes represent bacterial species and edges represent similarity between pairs of bacterial species based on gene family content, quantified using the 3-way Sørensen Index. Nodes are coloured according to genus. The same node colour key as in Fig. 2 applies.

Networks were also constructed using the standard 2-way Sørensen Index and pruned using a best edge approach and a Maximum Spanning Tree (MST) approach. For the best edge approach, the best and second best edges were selected for each node. The resulting network is shown in Fig. 3a.

A Maximum Spanning Tree is a useful approach for sparsifying a network by isolating the ‘backbone’ of the network as the shortest tree spanning all nodes which has maximum weight. The Sørensen MST can be seen in Fig. 3b.

The 3-way networks in Fig. 2 and S1 Fig. have an interesting structure. In each network, nodes of the same colour group together, indicating that the genera group together well. The network shown in Fig. 2 especially seems to show an interesting middle ground between connectedness and modularity. There are generally many connections within genera, but also some connections between genera. In contrast to this is the 2-way Sørensen MST shown in Fig. 3b. MSTs, by there very nature, have no modularity. This is clear in Fig. 3b where the genera do seem to group together, but there are no connections within the genera. MSTs thus give limited information, and should be used in combination with other types of networks and pruning methods. The 2-way Sørensen best edge network (Fig. 3a) was constructed by selecting only the best and second best edges for each node from the standard 2-way Sørensen network. It would appear that this 2-way best edge network is overly sparse, and does not give much information about the connectedness between genera. It would seem that the genera are also not as well grouped as in the 3-way best-edge network.

#### 3-way and 2-way Czekanowski networks

A new 3-way metric was developed called the 3-way Czekanowski Index. It is an extension of the standard 2-way Czekanowski Index [16] in the same way that the 3-way Sørensen Index [5] is an extension of the original 2-way Sørensen Index [15]. A 3-way network was constructed using the 3-way Czekanowski Index and pruned in the same way described above for the 3-way Sørensen network. The thresholded 3-way Czekanowski network and the best-edge 3-way Czekanowski network can be seen in S3 and 4 Figs. respectively. Networks were also constructed using the standard 2-way Czekanowski Index and can be seen in Figs. 5a and 5b.

**Fig 4:**
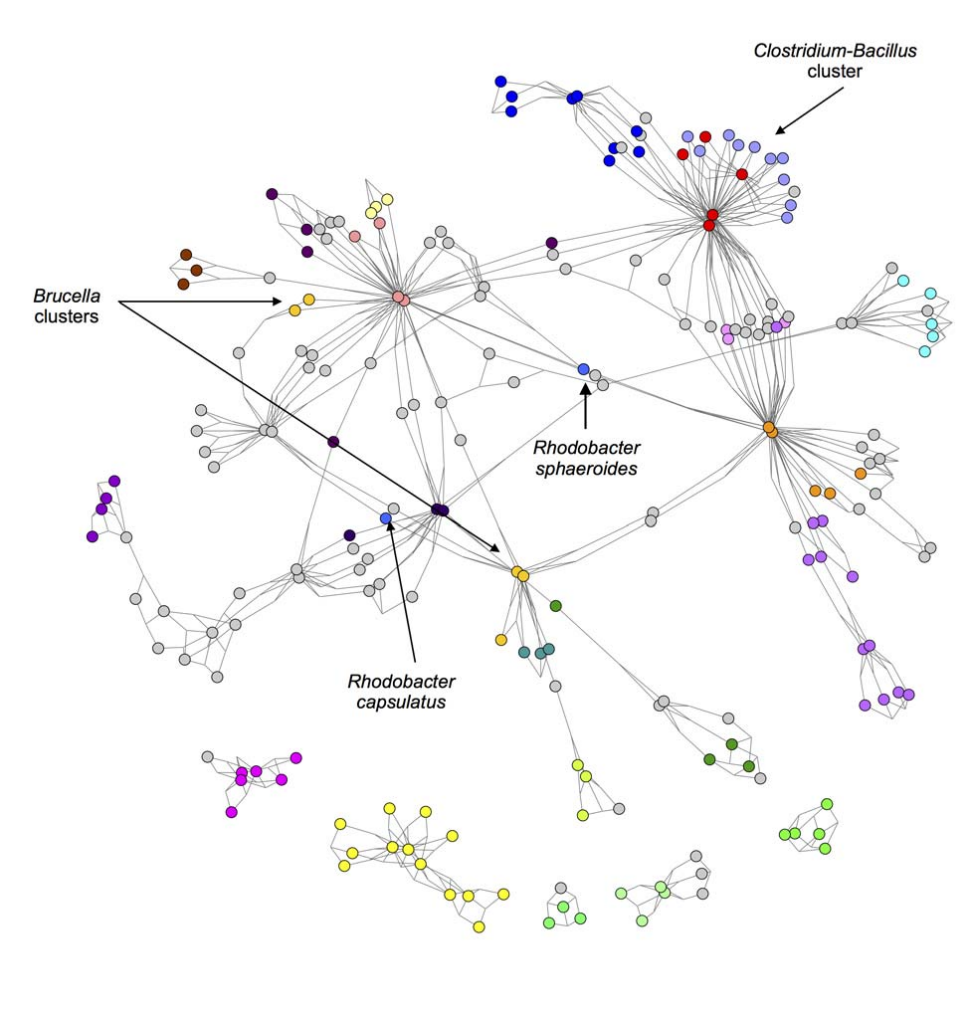
Best-Edges 3-way Czekanowski Network. 3-way Czekanowski network pruned by selecting the best and second best edge for each node. Nodes represent bacterial species and edges represent similarity between triplets of bacterial species based on gene family content, quantified using the 3-way Czekanowski Index. Nodes are coloured according to genus. The same node colour key as in Fig. 2 applies.

**Fig 5:**
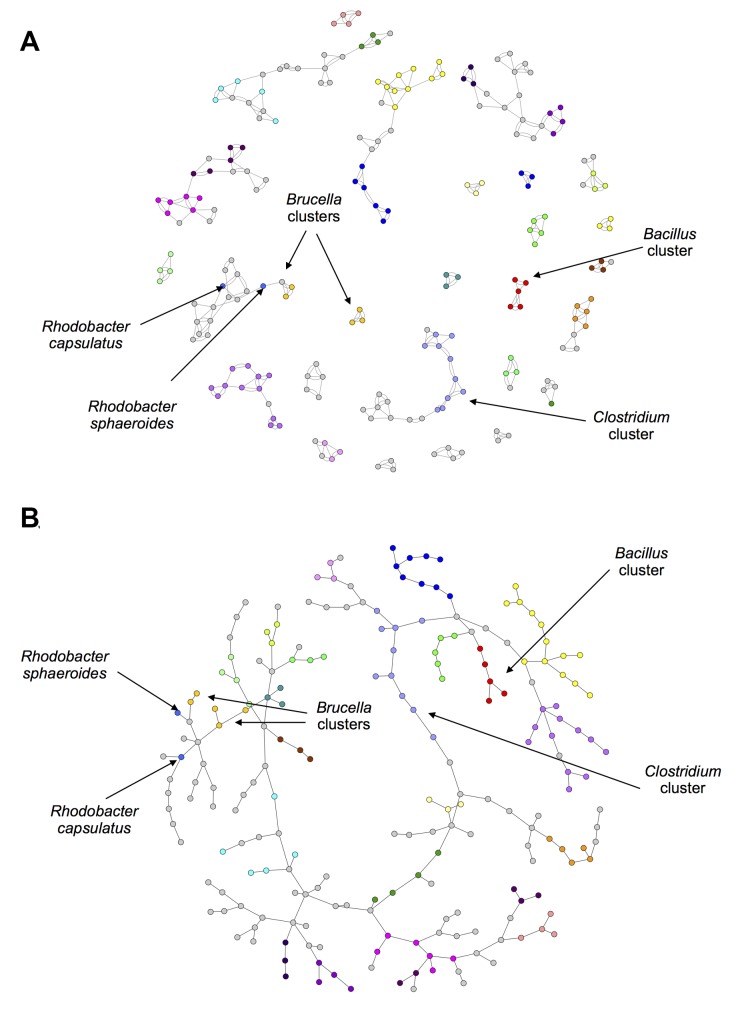
2-way Czekanowski Networks. (a) 2-way Czekanowski Best Edges Network (b) Maximum Spanning Tree (MST) of the all-vs-all Czekanowski network. 3-way Sørensen network pruned by selecting the best and second best edge for each node. Nodes represent bacterial species and edges represent similarity between pairs of bacterial species based on gene family content, quantified using the 3-way Sørensen Index. Nodes are coloured according to genus. The same node colour key as in Fig. 2 applies.

#### Gene family enrichment networks

**Fig 6:**
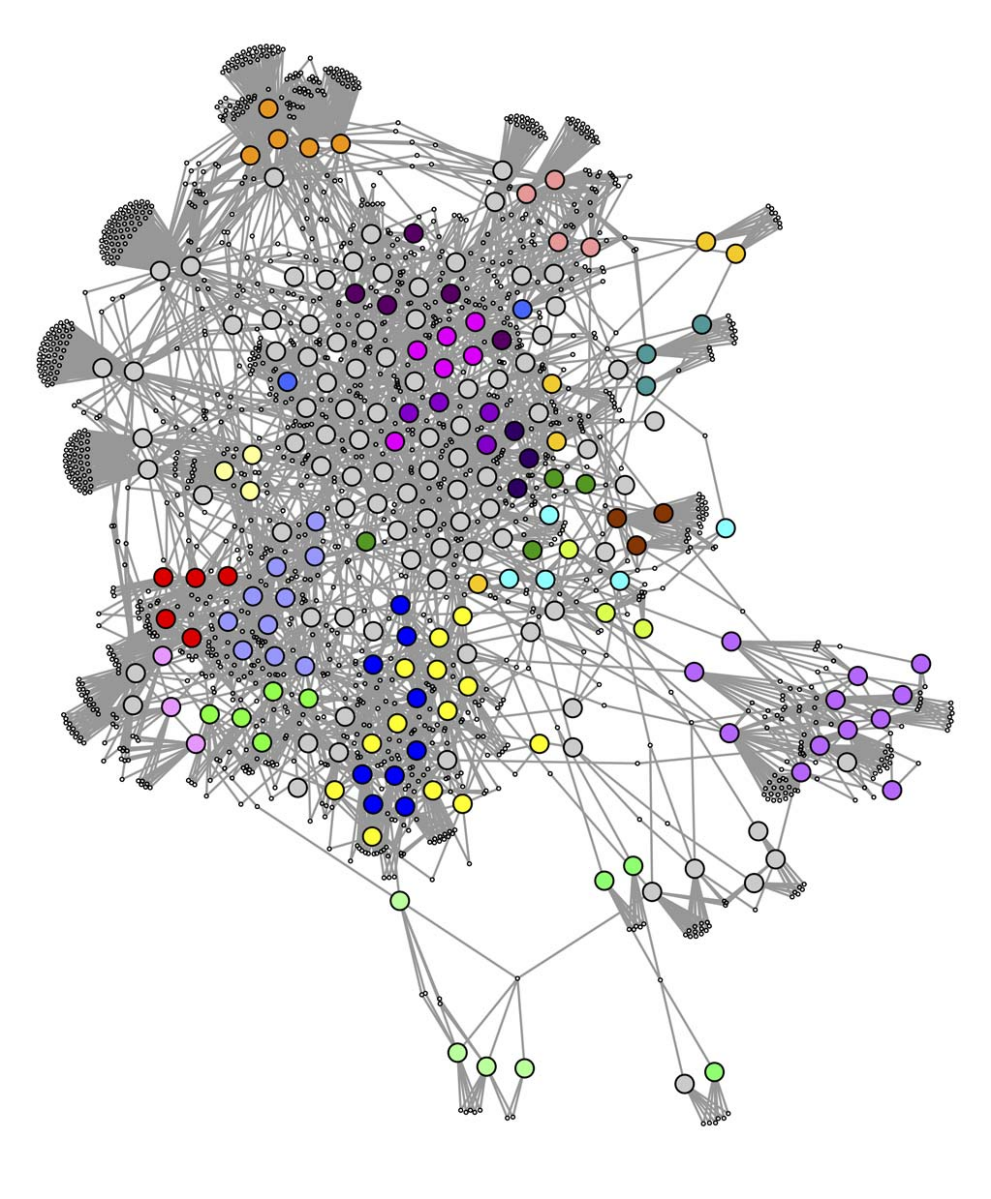
Shared enriched families. Network of bacteria species connected through shared enriched gene families. Small, white nodes represent gene families, coloured nodes represent bacterial species coloured by genus. Edges connect gene families to species in which they are enriched.

In order to get another perspective on the relationships between the bacteria species based on gene families, a gene family enrichment network was constructed (Fig. 6). In this network, large, coloured nodes represent bacterial species and small white nodes represent gene families which are enriched in more than one species as determined using Fisher’s Exact Test [18] Each gene family node is connected to the species in which the gene family is enriched. It can clearly be seen that the genera group together well in this network. Shared enriched families thus seem to be a competent measure of species similarity. This network also allows us to target gene families which seem to be distinguishing characteristics of small groups of species.

#### Network comparison

The 3-way Sørensen networks often support the interpretations of the 2-way networks. However, in some cases, the 3-way networks give new information which differs from that of the 2-way networks. A selection of examples have been selected in order to illustrate situations where the 3-way networks differ from 2-way networks, as well as examples where there is agreement between 2-way and 3-way networks. A procedure was implemented to calculate a “measure of disagreement” between the local topologies of genera between 2-way and 3-way networks. The number of edges within and between genera were quantified for each genera in 2-way and 3-way networks by counting the number of inbound edges (edges connecting species within genera) and outbound edges (edges connecting species across genera). A ratio of inbound over outbound edges was then calculated for each genera in 2-way networks and in 3-way networks, as well as the reciprocal ratio. These values were ranked for each network, and the differences between the rank of a genera’s ratio between the two types of networks were calculated. This was performed for both orientations of the ratio. These scores (see Supplementary S1 Table) give an indication of how different the modularity of a genus is between 2-way and 3-way networks, with larger values indicating a larger difference. The examples chosen to illustrate differences between the two types of networks did indeed have high scores.

#### *Clostridium-Bacillus* cluster

**Fig 7:**
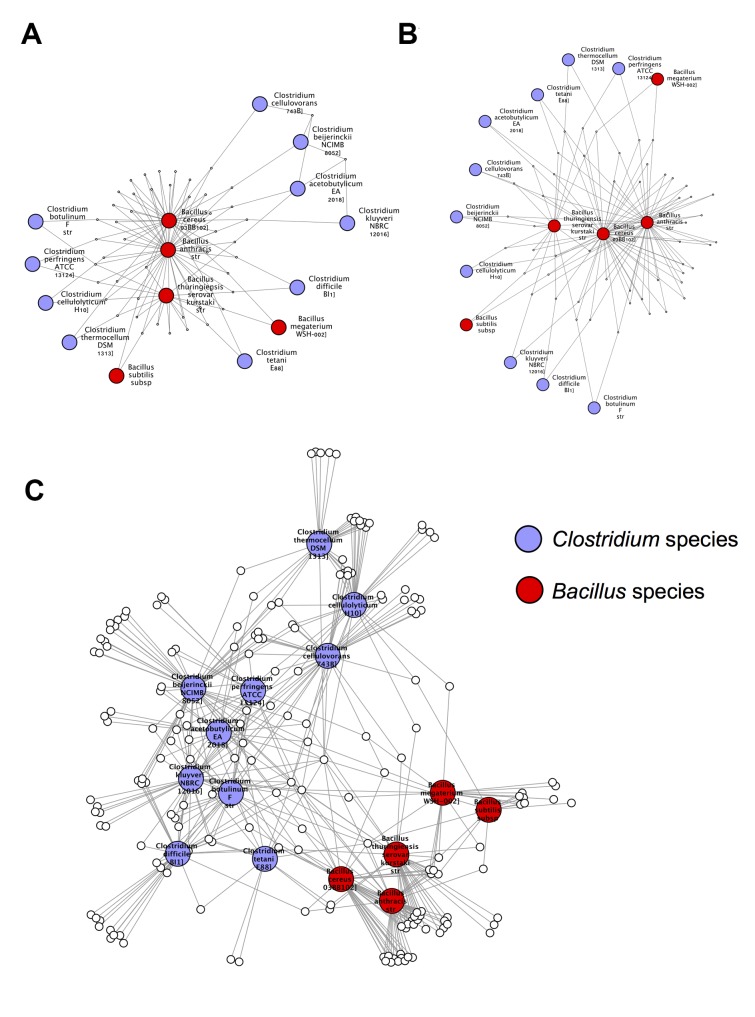
Clostridium and Bacillus subnetwork. Subnetworks containing the *Clostridium* and *Bacillus* species selected from (a) 3-way best edge Sørensen Network (b) 3-way best edge Czekanowski Network (c) Gene family enrichment network.

The cluster of red and light blue nodes in the 3-way Sørensen network (Fig. 2) and the 3-way Czekanowski network (Fig. 4) consist of *Clostridium* species (light blue nodes) and *Bacillus* species (red nodes). Fig. 7a and 7b show subnetworks containing these two clusters, and it is clear that, in both the Sørensen 3-way network and the Czekanowski 3-way network, there are a number of 3-way edges connecting species within and between those two genera. When looking at the same two genera in the 2-way Sørensen and 2-way Czekanowski networks (Figs. 3a, 3b, 5a and 5b) there is no evidence of any particular link between these 2 genera. In the 2-way Sørensen MST (Fig. 3b) the two genera are close together, but there are no edges between them. In the 2-way best edge Sørensen network (Fig. 3a) these two genera are in two completely separate modules, giving no indication whatsoever that they are connected or similar. Similar patterns are seen in the 2-way Czekanowski MST (Fig. 5b) and the 2-way best edge Czekanowski network (Fig. 5a). When looking at the shared enriched gene family network (Fig. 6) the *Clostridium* and *Bacillus* species are topologically close together. The *Clostridium* and *Bacillus* species as well as their neighbouring gene families were selected as a subnetwork from the family enrichment network and can be seen in Fig. 7c. It is apparent that the *Clostridium* and *Bacillus* species share several enriched gene families. The 3-way Sørensen and 3-way Czekanowski networks seem to be picking up a relationship between the two genera which is not seen in the 2-way networks, which is further supported by the gene family enrichment data.

Gene families which were enriched in both genera, and present in at least 3 species were selected for further analysis. The genes in these gene families were then compared against all *Clostridium* and *Bacillus* proteins in NCBI using BLAST [19, 20]. Many of the genes identified were related to sporulation. *Clostridium* and *Bacillus* species are known to sporulate and there is literature evidence for the conservation of various sporulation genes across these two genera [21]. Sporulation is a process which involves the production of a endospores, which are dormant and highly resistent to environmental stresses [21]. Examples of genes in these gene families enriched in both *Bacillus* and *Clostridium* species were AbrB and GerKA, which are known to be involved in sporulation in *Bacillus* species [22].

Another gene family enriched in both *Clostridium* and *Bacillus* species contained genes with polysaccharide deacetylase functions, in particular, the gene pdaB. There is literature evidence for the requirement of polysaccharide deacetylases for sporulation in *Bacillus subtilis*, in which pdaB mutants were unable to properly maintain their spores in the later stages of sporulation [23]. The pdaA gene has also been found to be neccesary for spore germination in *B. subtilis* [24]. The enrichment of this family in both *Clostridium* and *Bacillus* species along with the other sporulation families could suggest a similar role of deacetylases in the sporulation of *Clostridium* species.

We also found that another gene family enriched in both *Bacillus* and *Clostridium* species contained genes related to chemotaxis, namely a methyl accepting chemotaxis protein. Chemotaxis and sporulation are oppositely regulated processes and are both regulated by the major sporulation regulating protein Spo0A [25]. Thus, it would appear that even though *Bacillus* and *Clostridium* are quite distant phylogenetically, they share a set of sporulation related families which appear to be detected by 3-way networks, and are missed by simpler 2-way networks quantifying only 2-way relationships.

#### *Brucella* partitioning

**Fig 8:**
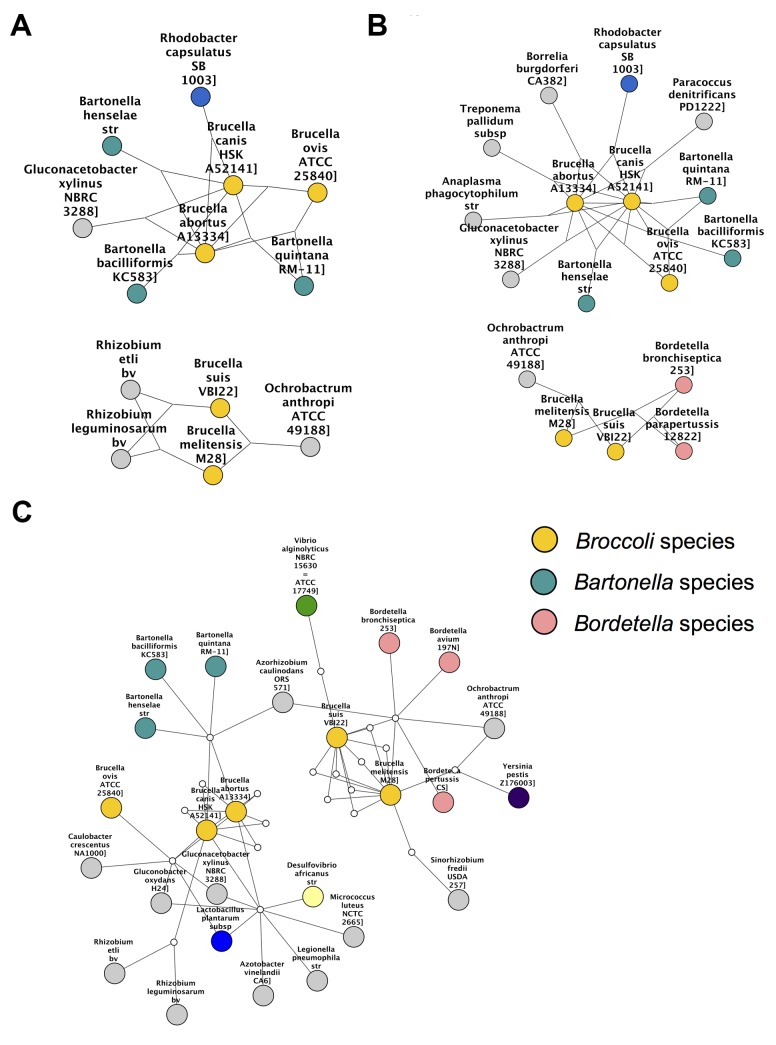
Clustering within *Brucella* genus. Subnetworks containing *Brucella* species constructed by selecting *Brucella* species and all neighbouring species nodes from (a) 3-way best edge Sørensen Network (b) 3-way best edge Czekanowski Network (c) Gene family enrichment network.

Species in the genus *Brucella* can be found as light orange nodes. In the Sørensen MST and the Czekanowski MST (Figs. 3b add 5b respectively), this genus is split into two groups, one group containing *B. canis*, *B. abortis* and *B. ovis* (Group 1), and the other group containing *B. melitensis* and *B. suis* (Group 2). These same separate groupings are also seen in the best-edge 3-way Sørensen network (Fig. 2) and best-edge 3-way Czekanowski network (Fig. 4). Thus using different 2-way and 3-way similarity metrics, the *Brucella* species partition in the same way. Fig. 8a and b show the neighbourhoods within one 3-way edge of the *Brucella* species in the best edge Sørensen network and the best edge Czekanowski network respectively. Fig. 8c is a subnetwork of the enrichment network (Fig. 6) showing all nodes within a radius of 2 of the *Brucella* nodes. From Fig. 8 the same groupings of the genus can be observed, thus this separation of the genus can be seen on a whole gene family profile scale, as well as on a gene family enrichment level. These groupings are different to the divergence previously found in the *Brucella* genus, in which *B. abortus* clustered nearer to *B. melitensis* and *B. suis* clustered nearer to *B. canis* [26].

From Fig. 8 a and b, it can be seen that both the 3-way Sørensen and 3-way Czekanowski networks group *Brucella ovis*, *Brucella canis* and *Brucella abortus* with members of the *Bartonella genus*. This is supported by the gene family enrichment view in Fig. 8c. Fig. 8a and b also suggests a relationship between Group 2 *Brucella* species and *Ochrobactrum anthropi*. This is also seen in the gene family enrichment view. Of the 3-way networks, only the Czekanowski network suggests that Group 2 of *Brucella* species, namely *Brucella suis* and *Brucella melitensis* group together with members of the *Bordetella* genus. This is also seen in the gene family enrichment view in Fig. 8c. None of the 2-way networks suggested this connection. The 2-way MSTs (Figs. 3b and 5b) show the proximity of Group 1 to the *Bartonella* species and the proximity of Group 2 to *O. anthropi*, however they do not suggest the link between Group 2 *Brucella* species and *Bordetella* species. The 2-way best edge networks (Figs. 3a and 5a) only show the connection between Group 2 and *O. anthropi*. They show none of the relationships suggested by 3-way networks between Group 1 and *Bartonella* species, and Group 2 and *Bordetella* species.

#### *Rhodobacter* separation

**Fig 9:**
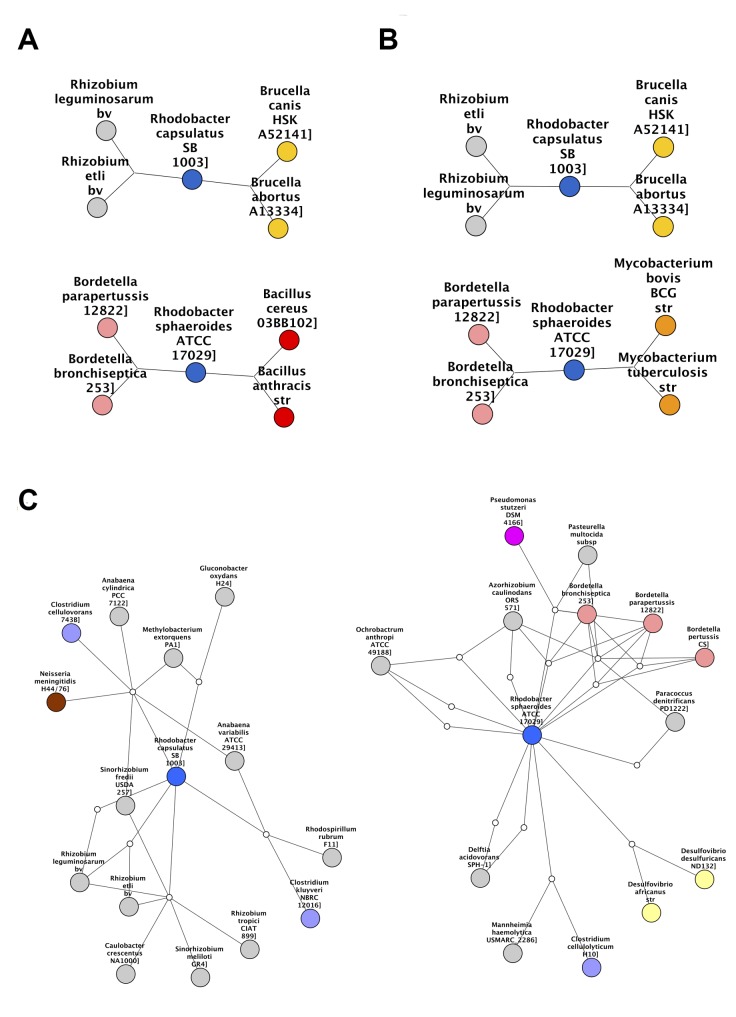
Separation of *Rhodobacter* species. Subnetworks containing *Rhodobacter* species constructed by selecting *Rhodobacter* species and all neighbouring species nodes from (a) 3-way best edge Sørensen Network (b) 3-way best edge Czekanowski Network (c) Gene family enrichment network.

Consider the genus *Rhodobacter* in the above networks (two medium blue nodes). In the Sørensen MST (Fig. 3b) these two nodes are neighbours. This is also seen in the best edge Sørensen network (Fig. 3a). However, in both Czekanowski 2-way networks (Figs. 5b and 5a), these two *Rhodobacter* species are not neighbours. The 3-way Sørensen and 3-way Czekanowski networks (Figs. 2 and 4) place these nodes quite far apart. Fig. 9a and b show the neighbourhoods within one 3-way edge of *Rhodobacter* species in the 3-way Sørensen network and 3-way Czekanowski network respectively. From this figure, it can be seen that the nodes are in separate neighbourhoods. This is also seen in the enriched family view in Fig. 9c. This figure shows the species which share at least one enriched family with *Rhodobacter* species. Both Sørensen and Czekanowski best edge 3-way networks thus pick up a separation between the two *Rhodobacter* species which is supported by the gene family enrichment data and not found by the 2-way Sørensen networks.

#### Combination view: *Rhodobacter* and *Brucella* species

**Fig 10:**
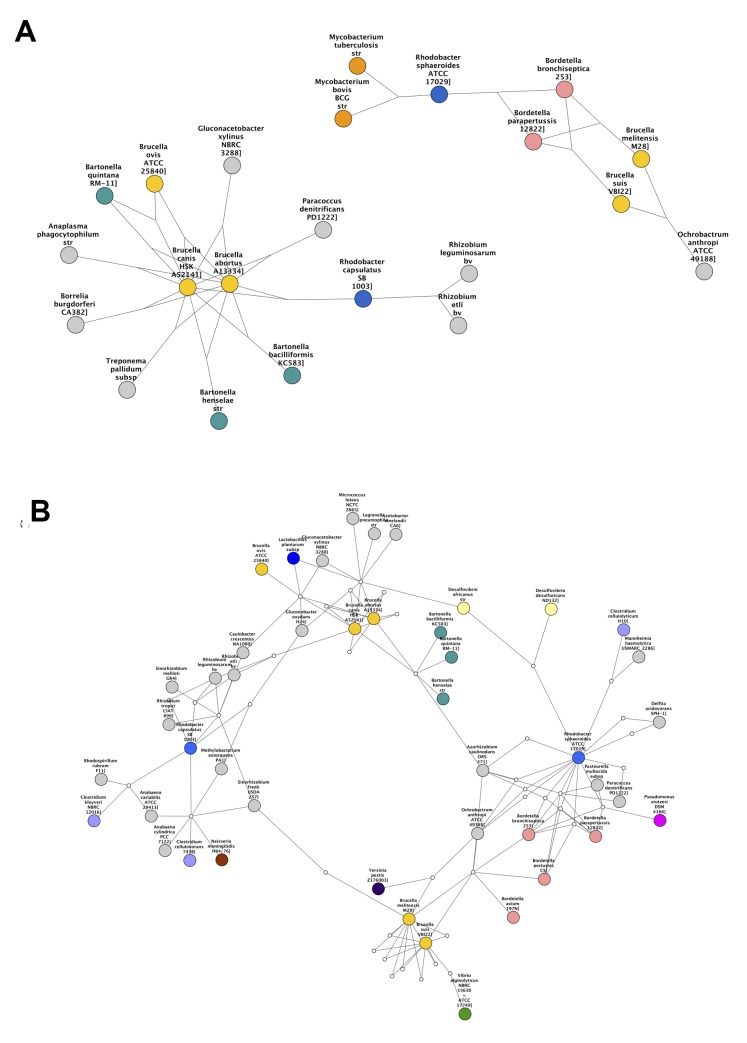
*Rhodobacter* and *Brucella* species. Subnetworks containing *Brucella* and *Rhodobacter* species constructed by selecting *Brucella* and *Rhodobacter* species and all neighbouring species nodes from (a) 3-way best edge Czekanowski Network (b) Gene family enrichment network.

A further examination of Figs. 8 and 9 shows that there seem to be overlaps between the *Brucella* groupings in Fig. 8 and the *Rhodobacter* groupings in Fig. 9. Fig. 10 shows the neighbourhood around *Brucella* species and *Rhodobacter species* in (a) the 3-way best edge Czekanowski network and (b) the gene family enrichment network. Group 1 *Brucella* species cluster with *Bartonella* species and *Rhodobacter capsulatus* and Group 2 *Brucella* species cluster witth *Bordetella* species, *Ochrobactrum athropi* and *Rhodobacter sphaeroides*. This amount of detail in groupings of species was not found in any of the 2-way networks.

#### Combined 2-way and 3-way networks

Merging the 3-way best edge Sørensen network (Fig. 2) and the 2-way Sørensen MST (Fig. 3b) results in an interesting network which is shown in S4 Fig. This network combines the modularity of the 3-way network showing the connections within genera and a few cross-genera connections with the MST which shows the overall connections across genera. This combined 2-way and 3-way Czekanowkski network (S5 Fig.) was also constructed by merging the 3-way best edge Czekanowski network (Fig. 4) and the 2-way Czekanowski MST (Fig. 5b). These combination networks provide an interesting, “best of both worlds” view. They combine the connectedness and simplicity of an MST, which allows for no modularity, but forces all nodes to connected to the network, and the modularity and complex relationships provided by the 3-way networks which show a mixture of within-module connection and inter-module connections, and show relationships missed by standard 2-way networks.

### Conclusions

3-way networks were explored for their use in comparative genomics and their utility in modelling more complex relationships. These networks, when used to model the phylogenomic relationships between 211 bacterial species revealed relationships between the species which were not found when using standard 2-way network models. These networks will be a useful tool for comparative genomics in order to model and reveal complex relationships.

## Materials and Methods

### Bacterial Gene Family Construction

Gene families were constructed using the TribeMCL pipeline [17]. An all-vs-all protein BLAST [19] was performed on the translated genomes of 211 bacterial species to calculate the sequence similarity between all pairs of proteins across the 211 bacterial genomes. An E-value cutoff of 10^−5^ was used. The Perl script orthomclBlastParser from the OrthoMCL package [27] was then used to parse the Blast results in order to select only the best Blast match per gene pair. For each gene pair *ab*, a score *S*_*ab*_ was calculated as [17]:

$$\begin{array}{c} S_{ab}=\log_{2}\left(\frac{E_{ab}+E_{ba}}{2}\right) \end{array}$$ (11)

where *E*_*ab*_ and *E*_*ba*_ are the E-values for the reciprocal BLAST hits between gene *a* and gene *b*. This resulted in a network in which each node represented a gene and each edge *ab* represented the similarity between the two nodes (*a* and *b*) which it connects, weighted by the similarity score *S*_*ab*_. MCL was then applied using an inflation value of 2 to cluster the network into gene families [28]. From the resulting gene families, a matrix was constructed called the Species-Family (SF) matrix, in which the rows represented bacterial gene families constructed using TribeMCL, and columns represented bacterial species, and each entry *ij* represented the number of genes in gene family *i* present in species *j*.

### 3-way Network Construction

The 3-way Sørensen Index and the 3-way Czekanowski Index was used to quantify the similarity between each triplet of species. Let *X*_*i*_ and *Y*_*i*_ and *Z*_*i*_ represent the *i* ^*th*^ element in columns *X*, *Y* and *Z* of the SF-matrix (i.e. the number of members of gene family *i* in species *X* species *Y* and species *Z* respectively. Let *X*_*B*_, *Y*_*B*_ and *Z*_*B*_ be the binary vectors associated with vectors *X*, *Y* and *Z* respectively. For each triplet of species (*X*, *Y*, *Z*) the Sørensen Index was calculated using Equation 7 and the Czekanowski Index was calculated using Equation 9. This resulted in a Sørensen 3-way network and a Czekanowski 3-way network. Using Theorem 1, any threshold set above 0.75 will exclude any 3-way relationships with no 3-way intersection contribution. Thus, a threshold of 0.76 was applied to each network and visualized in Cytoscape [29] using an Allegro layout. These networks can be seen in S1 and S2 Figs. Cytoscape can only visualize 2-way networks in the sense that it can only handle edges connecting 2 nodes. To our knowledge, no visualization software exists for 3-way networks. Thus, the 3-way network had to be transformed such that it could be visualized in Cytoscape. To do so, each 3-way-edge was represented by a node with degree 3, connected to the bacterial species nodes which the 3-way-edge connected. In the transformed network, each node thus either represented a bacterial species or a 3-way edge (referred to as an ‘edge-node’). A close-up of these 3way-edges can be seen in S3 Fig.

A best-edge approach was also used to prune the 3-way networks. For each bacterial species node, the best and second best edges (edges with the highest and second highest weight) were selected. A network was constructed and transformed into a format which can be visualized in Cytoscape as described above. The resulting networks can be seen in Figs. 2 and 4.

### 2-way Network Construction

The standard 2-way Sørensen and 2-way Czekanowski Indices were used to quantify the similarities between all pairs of species. Let *X*_*i*_ and *Y*_*i*_ represent the *i* ^*th*^ element in column *X* and column *Y* in the SF-matrix (i.e. the number of members of gene family *i* in species *X* and species *Y* respectively. Let *X*_*B*_ be the binary vector associated with vector *X* and *Y*_*B*_ be the binary vector associated with vector *Y*. For each pair of species (*X*, *Y*) the Sørensen Index was calculated using Equation 3 and the Czekanowski Index was calculated using Equation 8. These networks were pruned using two approaches, namely a Maximum Spanning Tree and best edge selection. The Maximum Spanning Tree was calculated by converting the network from a similarity network into a distance network by inverting the edge weights i.e. for each edge weight *w* the inverted edge weight *w*′ was calculated as

$$w^{'}=1-w.$$

A Minimum Spanning Tree algorithm was then applied to the distance network using the Dijkstra algorithm from the Graph Perl Module (Jarkko Hietaniemi, http://www.cpan.org/). For best edge selection, the best and second best edge for each node was selected based on edge weight. These pruned networks were visualized in Cytoscape [29] using an Allegro layout, and can be seen in Figs. 3a, 3b, 5a and 5b.

### Combined 2-way and 3-way Network Construction

For both the Sørensen Index and the Czekanowski Index, the union of the 3-way best-edge network and the 2-way MST was calculated, resulting in a combined network model. These can be seen in S4 and S5 Figs.

### Gene Family Enrichment

Fisher’s exact test [18], followed by Holm-Bonferroni multiple hypothesis correction [30] was used to determine enrichment of gene families within species. A p-value cutoff of 0.05 was used. Gene families which were enriched in more than one species (so-called shared-enriched families) were selected and a new network was constructed in which each node represented either a bacterial species or a gene family, and each edge connected a gene family to bacterial species in which it was enriched. The species were coloured according to their genera. The network was visualized in Cytoscape [29] using an Allegro layout (Fig. 6).

## Supporting Information

S1 Text NCBI IDs. NCBI IDs for each of the 211 bacterial genomes.
(PDF)

S1 Table Measure of disagreement. Difference between the ratios of inbound over outbound edges ($D_{o}^{i}$), as well as the difference between the reciprocal ratios ($D_{i}^{o}$) for each genus in the 2-way and 3-way best edge networks.
(PDF)

S1 File Cytoscape session. A Cytoscape session file containing the 2-way and 3-way networks.
(ZIP)

S1 Fig Thresholded 3-way Sørensen Network. Network constructed by setting a 0.76 threshold for the 3-way Sørensen Network, and removing all 3-way edges below this threshold.
(TIFF)

S2 Fig Thresholded 3-way Czekanowski Network. Network constructed by setting a 0.76 threshold for the 3-way Czekanowski Network, and removing all 3-way edges below this threshold.
(TIFF)

S3 Fig 3-way edges. Close-up of a section of the thresholded 3-way network showing the 3-way edges. Large, coloured nodes represent bacterial species, whereas small white nodes and their respective 3 edges represent 3-way edges connecting the bacterial nodes.
(TIFF)

S4 Fig Union Sørensen MST and Sørensen 3-way Best Edge Network. Network constructed by taking the union of the Sørensen 3-way Best Edge Network (Fig. 2) and the Sørensen MST (Fig. 3b).
(TIFF)

S5 Fig Union Czekanowski MST and Czekanowski 3-way Best Edge Network. Network constructed by taking the union of the Czekanowski 3-way Best Edge Network (Fig. 4) and the Czekanowski MST (Fig. 5b).
(TIFF)

S6 Fig Distributions. Distributions of the 2-way and 3-way similarity metrics.
(TIFF)
