# Supplementary material for: 3-way Networks: Application of Hypergraphs for Modelling Increased Complexity in Comparative Genomics
Source: PLoS Comput Biol. 2015 Mar 27;11(3):e1004079. doi: 10.1371/journal.pcbi.1004079 (PMC4376783; doi:10.1371/journal.pcbi.1004079)
Supplement: S1 Text — NCBI IDs for each of the 211 bacterial genomes. (PDF) [file pcbi.1004079.s001.pdf]

NC_000919
NC_002179
NC_002620
NC_002928
NC_002940
NC_002967
NC_002977
NC_003295
NC_003454
NC_003888
NC_004461
NC_004557
NC_004741
NC_005364
NC_005956
NC_006368
NC_007005
NC_007168
NC_007294
NC_007350
NC_007413
NC_007606
NC_007633
NC_008054
NC_008095
NC_008261
NC_008309
NC_008497
NC_008543
NC_008599
NC_008687
NC_008783
NC_008789
NC_009050
NC_009079
NC_009348
NC_009439
NC_009446
NC_009505
NC_009617
NC_009668
NC_009708
NC_009727
NC_009778
NC_009785
NC_009792
NC_009937
NC_010002
NC_010163
NC_010172
NC_010572
NC_010645
NC_010658
NC_010688
NC_010793
NC_010939
NC_011312
NC_011374

NC_011420
NC_011837
NC_011896
NC_011898
NC_011916
NC_012026
NC_012039
NC_012468
NC_012472
NC_012779
NC_012803
NC_012850
NC_012857
NC_013446
NC_013511
NC_013716
NC_013853
NC_013928
NC_013948
NC_013949
NC_013961
NC_014029
NC_014033
NC_014034
NC_014121
NC_014370
NC_014393
NC_014623
NC_014659
NC_014752
NC_014810
NC_014920
NC_014921
NC_014965
NC_015291
NC_015381
NC_015571
NC_015663
NC_015726
NC_015964
NC_016027
NC_016445
NC_016603
NC_016629
NC_016775
NC_016776
NC_016778
NC_016795
NC_016802
NC_016803
NC_016804
NC_016822
NC_016829
NC_016845
NC_016915
NC_017066
NC_017138
NC_017171

NC_017179
NC_017223
NC_017245
NC_017272
NC_017289
NC_017295
NC_017297
NC_017304
NC_017309
NC_017310
NC_017353
NC_017456
NC_017462
NC_017474
NC_017491
NC_017504
NC_017511
NC_017516
NC_017530
NC_017532
NC_017551
NC_017563
NC_017564
NC_017566
NC_017582
NC_017584
NC_017632
NC_017671
NC_017729
NC_017764
NC_017831
NC_017861
NC_017953
NC_018000
NC_018077
NC_018080
NC_018106
NC_018108
NC_018221
NC_018413
NC_018498
NC_018513
NC_018521
NC_018525
NC_018533
NC_018608
NC_018646
NC_018690
NC_018707
NC_019382
NC_019396
NC_019551
NC_019771
NC_019845
NC_019966
NC_019971
NC_020059
NC_020207

NC_020211
NC_020238
NC_020418
NC_020450
NC_020507
NC_020508
NC_020526
NC_020800
NC_020992
NC_021150
NC_021151
NC_021181
NC_021200
NC_021214
NC_021224
NC_021235
NC_021237
NC_021290
NC_021352
NC_021521
NC_021742
NC_021744
NC_021807
NC_021824
NC_021831
NC_021872
NC_021881
NC_021883
NC_021905
NC_022000
NC_022048
NC_022244
NC_022245
NC_022246
NC_022347
NC_022349
NC_022350
