# Supplementary material for: 3-way Networks: Application of Hypergraphs for Modelling Increased Complexity in Comparative Genomics
Source: PLoS Comput Biol. 2015 Mar 27;11(3):e1004079. doi: 10.1371/journal.pcbi.1004079 (PMC4376783; doi:10.1371/journal.pcbi.1004079)
Supplement: S1 Table — Difference between the ratios of inbound over outbound edges (Doi), as well as the difference between the reciprocal ratios (Dio) for each genus in the 2-way and 3-way best edge networks. (PDF) [file pcbi.1004079.s002.pdf]

Table S1: Measure of disagreement: Difference between the ratios of inbound over outbound edges (D_o^i), as well as the difference between the reciprocal ratios (D_i^o) for each genus in the 2-way and 3-way best edge networks.

Genus	D_o^i	D_i^o
Ochrobactrum	0	6
Haemophilus	0	3
Orientia	1	5
Campylobacter	1	3
Edwardsiella	1	1
Cronobacter	2	4
Coxiella	2	4
Francisella	2	4
Salmonella	2	4
Micrococcus	2	4
Methylobacterium	2	4
Myxococcus	2	4
Gluconacetobacter	2	4
Azorhizobium	2	4
Pasteurella	2	4
Erwinia	2	4
Acholeplasma	2	4
Ralstonia	2	4
Thermus	2	4
Vibrio	2	2
Brucella	2	3
Shigella	3	3
Ureaplasma	3	3
Delftia	3	3
Listeria	3	3
Mannheimia	3	3
Methylococcus	3	3
Prevotella	3	2
Corynebacterium	4	2
Yersinia	4	2
Propionibacterium	4	2
Bacteroides	4	2
Rhodococcus	4	2
Stigmatella	4	2
Shewanella	4	2
Borrelia	4	2
Paracoccus	4	2
Halorhodospira	4	2
Escherichia	4	2

Gardnerella	4	2
Comamonas	4	2
Lactococcus	4	2
Chlamydophila	4	2
Thermotoga	4	2
Taylorella	4	2
Stenotrophomonas	4	2
Azotobacter	4	2
Dichelobacter	4	2
Morganella	4	2
Mycoplasma	4	4
Aliivibrio	4	2
Fusobacterium	4	2
Gluconobacter	4	2
Porphyromonas	4	2
Caulobacter	4	2
Cupriavidus	4	2
Pectobacterium	4	2
Legionella	4	2
Leptospira	4	2
Rickettsia	4	6
Anaplasma	5	8
Mycobacterium	5	5
Bordetella	5	9
Lactobacillus	5	4
Citrobacter	5	57
Neisseria	6	6
Staphylococcus	6	1
Burkholderia	6	5
Proteus	6	0
Streptomyces	7	7
Acinetobacter	7	6
Serratia	7	7
Rhizobium	7	7
Klebsiella	7	7
Streptococcus	9	10
Chlamydia	9	9
Sinorhizobium	10	7
Enterobacter	10	10
Bacillus	10	6
Pseudomonas	11	10
Helicobacter	14	17
Actinobacillus	14	13
Xanthomonas	15	21

Anabaena	15	61
Desulfovibrio	15	16
Aeromonas	19	17
Clostridium	24	24
Bartonella	26	26
Treponema	34	28
Enterococcus	43	24
Rhodobacter	50	3
Rhodospirillum	50	4
