# Supplementary figures and images for: 3-way Networks: Application of Hypergraphs for Modelling Increased Complexity in Comparative Genomics
Source: PLoS Comput Biol. 2015 Mar 27;11(3):e1004079. doi: 10.1371/journal.pcbi.1004079 (PMC4376783; doi:10.1371/journal.pcbi.1004079)

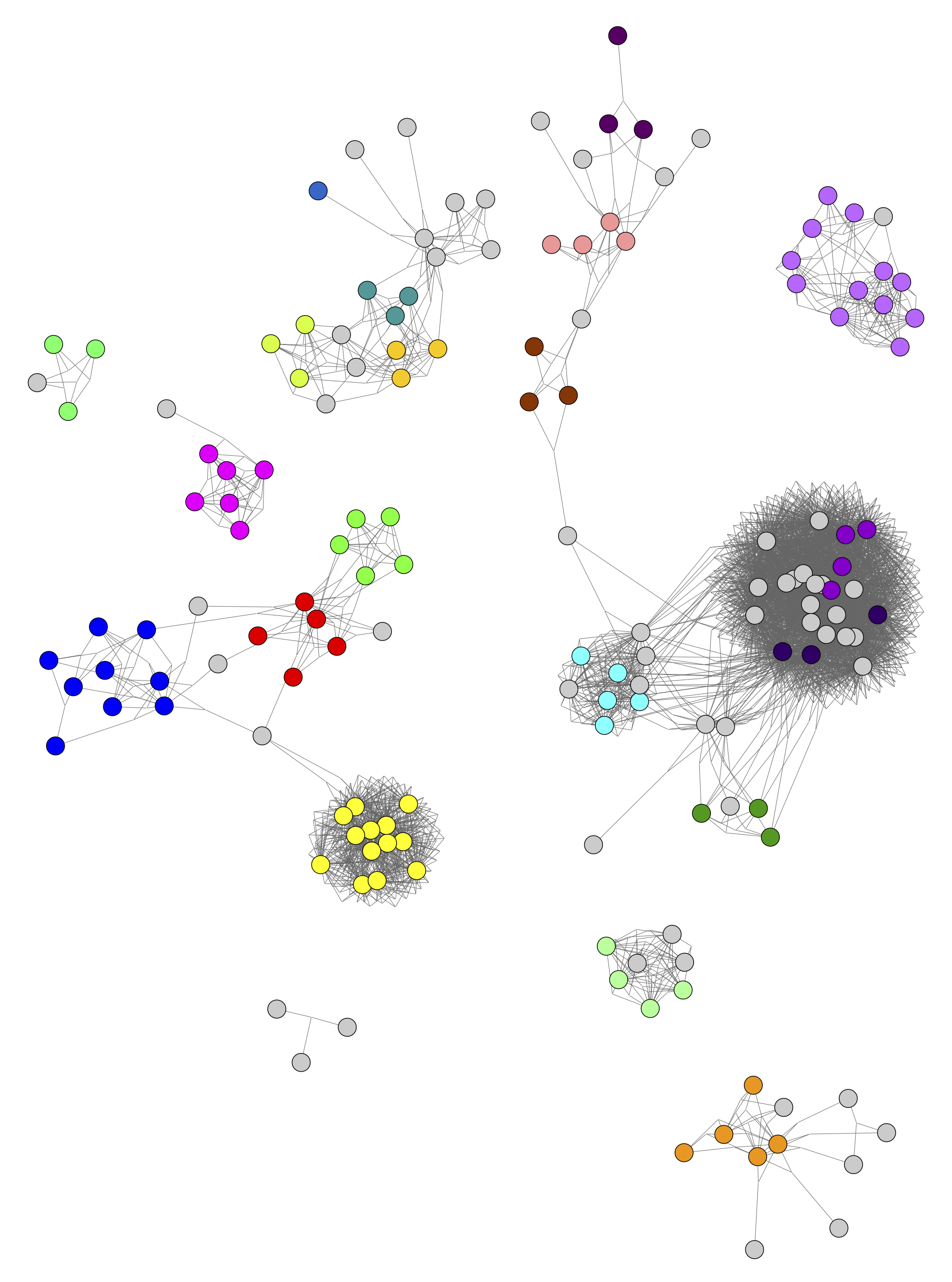

Supplement: S1 Fig — Network constructed by setting a 0.76 threshold for the 3-way Sørensen Network, and removing all 3-way edges below this threshold. (TIFF) [file pcbi.1004079.s004.tiff]

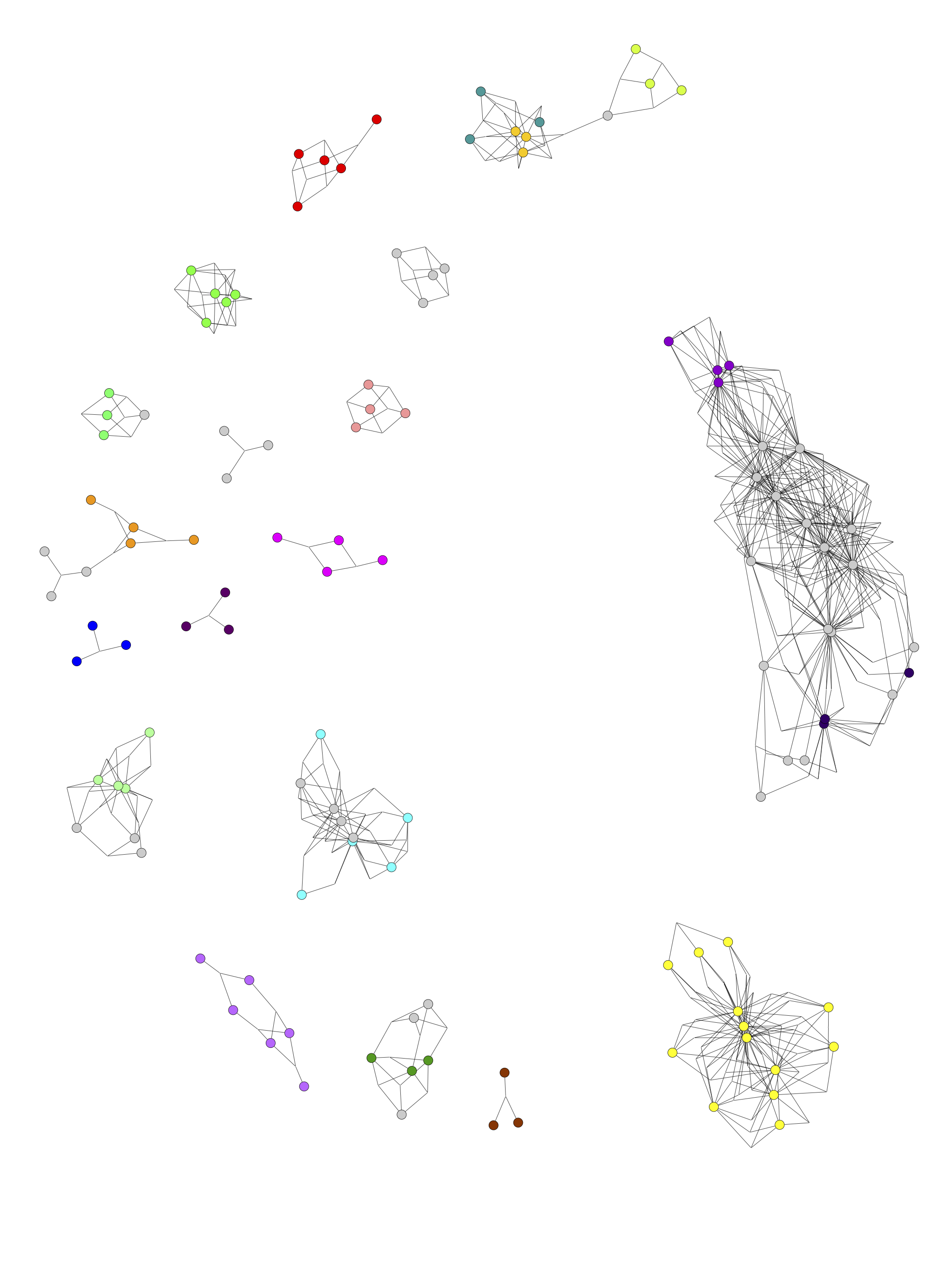

Supplement: S2 Fig — Network constructed by setting a 0.76 threshold for the 3-way Czekanowski Network, and removing all 3-way edges below this threshold. (TIFF) [file pcbi.1004079.s005.tiff]

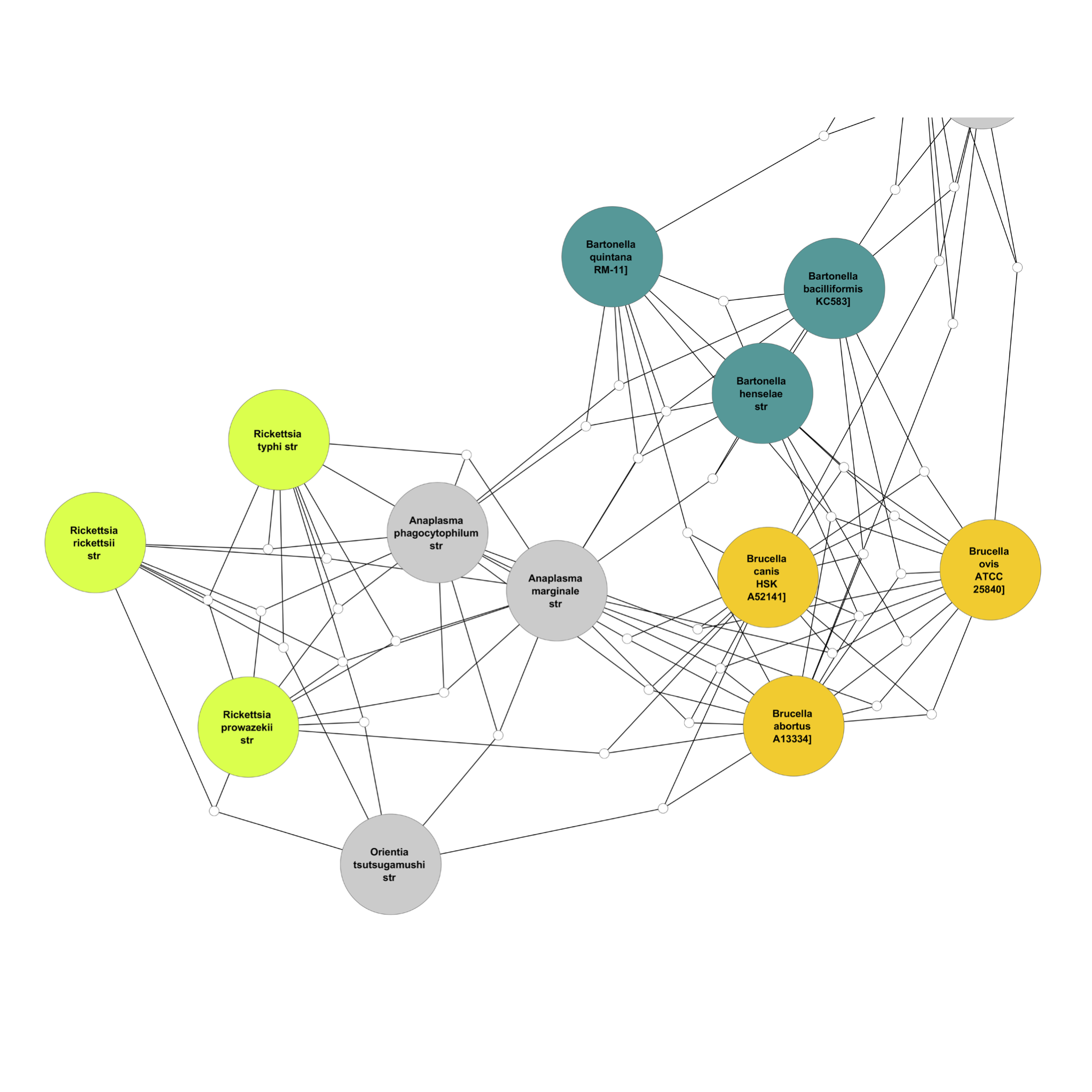

Supplement: S3 Fig — Close-up of a section of the thresholded 3-way network showing the 3-way edges. Large, coloured nodes represent bacterial species, whereas small white nodes and their respective 3 edges represent 3-way edges connecting the bacterial nodes. (TIFF) [file pcbi.1004079.s006.tiff]

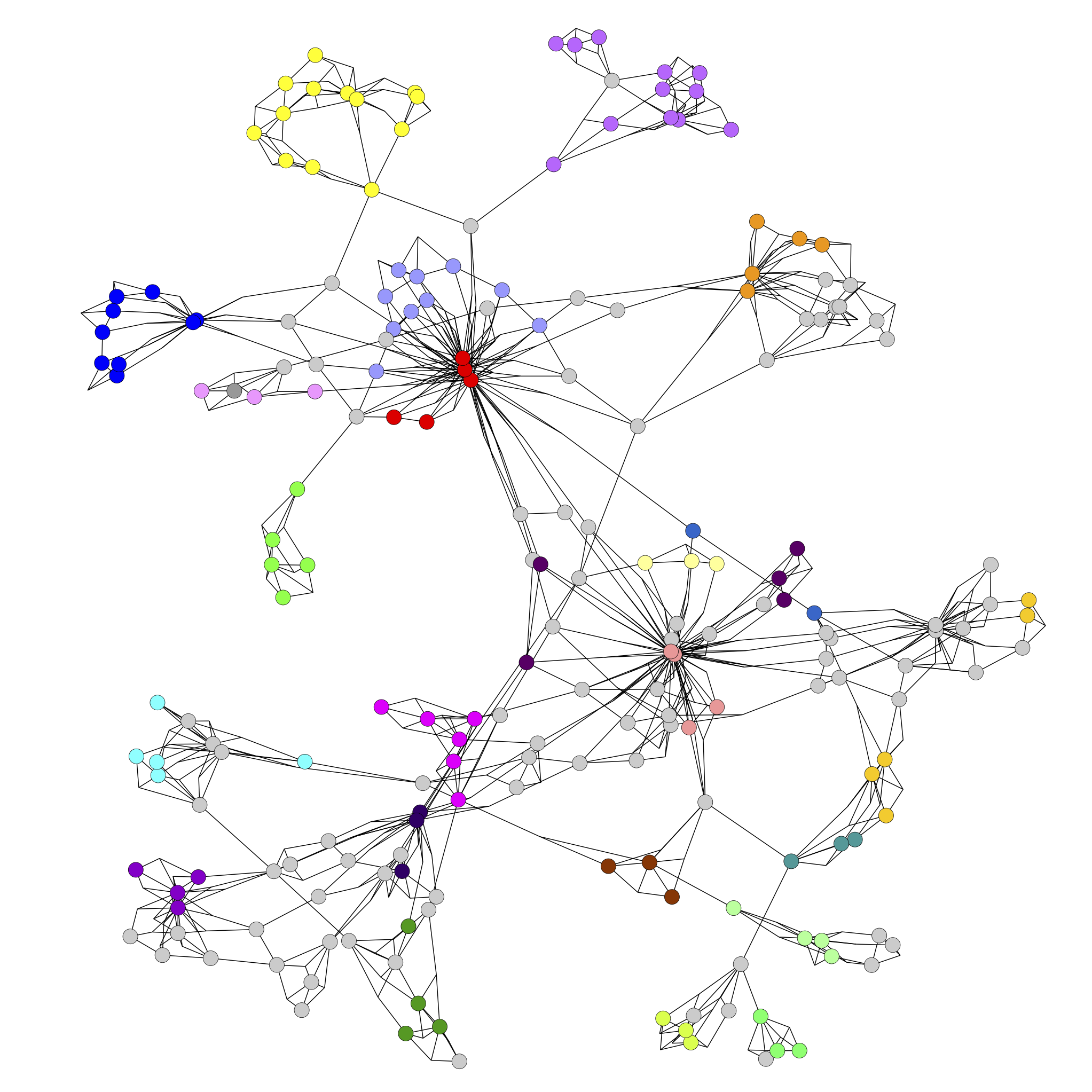

Supplement: S4 Fig — Network constructed by taking the union of the Sørensen 3-way Best Edge Network (Fig. 2) and the Sørensen MST (Fig. 3b). (TIFF) [file pcbi.1004079.s007.tiff]

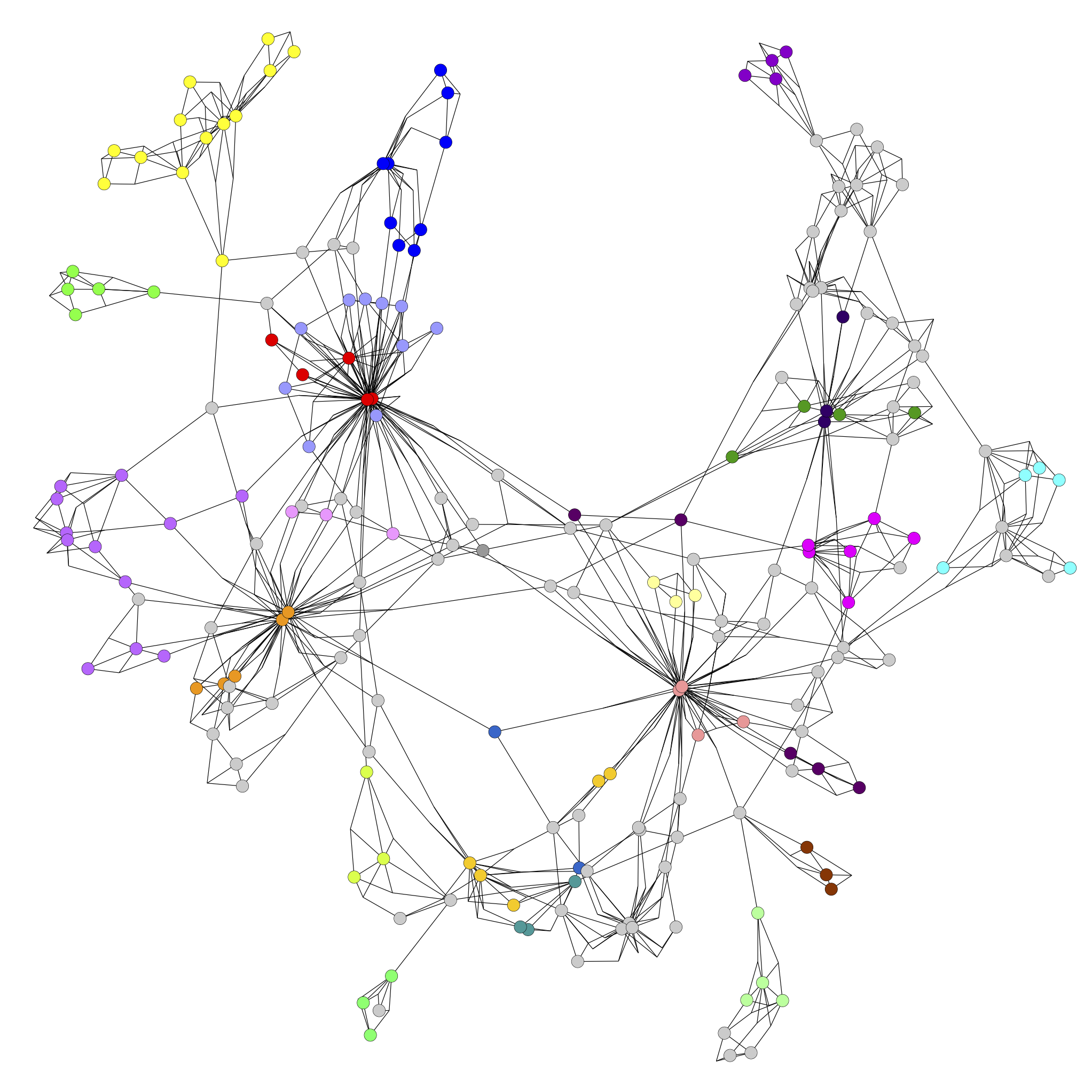

Supplement: S5 Fig — Network constructed by taking the union of the Czekanowski 3-way Best Edge Network (Fig. 4) and the Czekanowski MST (Fig. 5b). (TIFF) [file pcbi.1004079.s008.tiff]

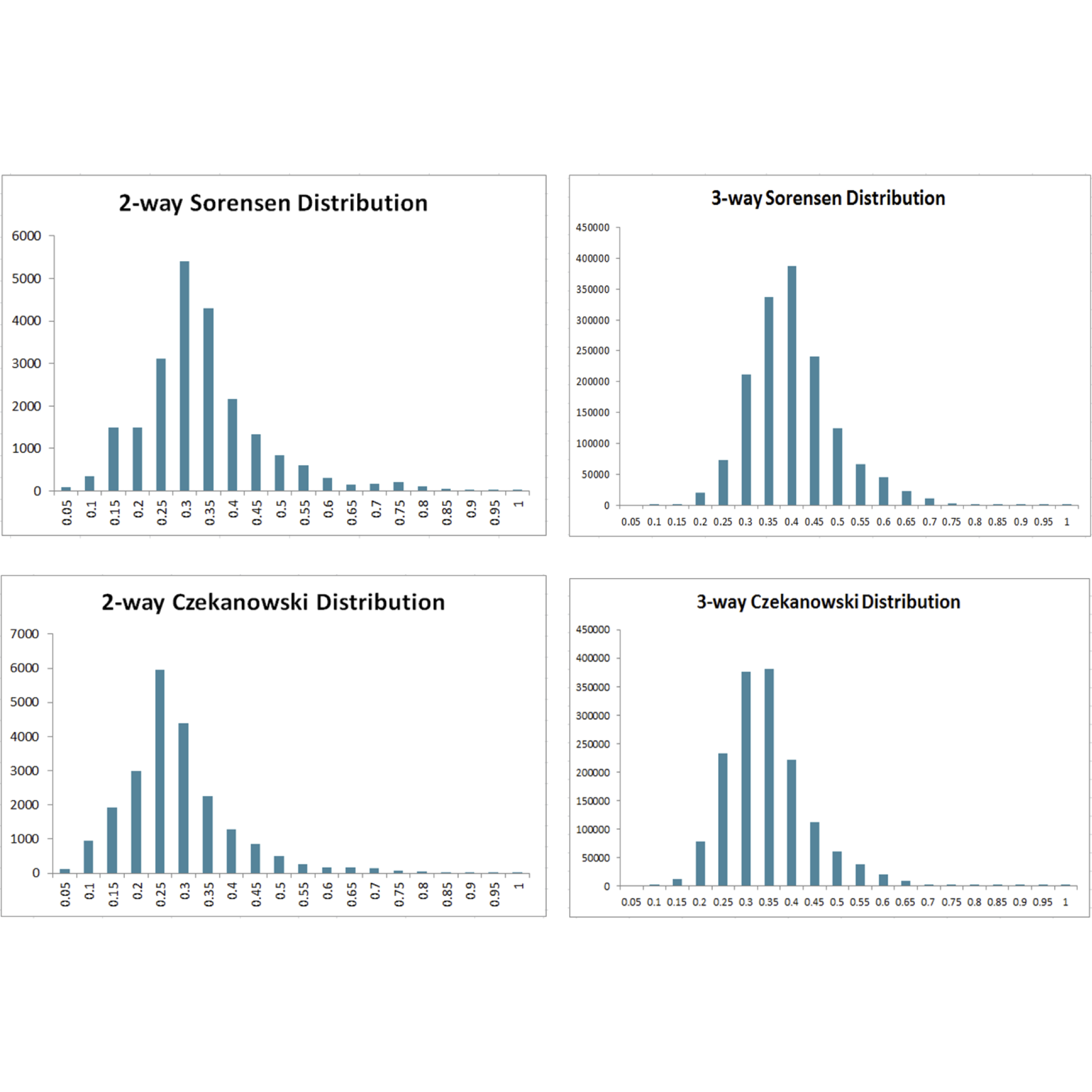

Supplement: S6 Fig — Distributions of the 2-way and 3-way similarity metrics. (TIFF) [file pcbi.1004079.s009.tiff]
